# Evaluation of paediatric liaison psychiatry services in England 2015-2019

**DOI:** 10.1192/bjo.2021.856

**Published:** 2021-06-18

**Authors:** Declan Hines, William Lee, Tamsin Ford, Sophie Westwood

**Affiliations:** 1 School of Clinical Medicine, University of Cambridge; 2 Hon Associate Professor, University of Exeter and NHS Liaison for PenCLAHRC; 3 Department of Psychiatry, University of Cambridge; 4 University of Plymouth

## Abstract

**Aims:**

Liaison psychiatry services (LPSs) provide psychiatric care to general medical patients. This paper aims to evaluate LPS provision for children and young people In England.

**Method:**

The annual Liaison Psychiatry Surveys of England (LPSEs) included questions on paediatric services from 2015 (LPSE-2). Questions were developed in consultation with NHS England and the Liaison Faculty of the Royal College of Psychiatrists. We analysed data from LPSE-2 and three subsequent surveys.

LPSs were systematically identified by contacting all acute hospitals with Type 1 emergency departments listed by NHS England. All identified LPSs were emailed a copy of the questionnaire, with follow-up emails and telephone contact for non-responders. Responses by email, post or telephone were accepted.

**Result:**

The number of acute hospitals with access to paediatric LPSs increased from 29 (16%) in 2015 to 46 (27%) in 2019; all of these hospitals had access to adult LPSs. The number of paediatric LPSs with at least 11 full time equivalent (FTE) mental health practitioners (MHPs) has increased from 6% to 24% and from none to 16% with 13 FTE or more MHPs. For both LPSE-4 and LPSE-5, there were only two acute hospitals where both 8 FTE MHPs and 1.5 FTE consultants were present. For LPSE-4, only one site met the Core 24 criteria (for adults - there are no criteria for paediatric LPSs) of 11 FTE MHPs and 1.5 FTE consultants, and for LPSE-5, both these sites exceeded them. Other paediatric services did not meet the adult core 24 criteria for a LPS.

Acute hospitals with access to 24/7 paediatric LPSs increased from 12% to 19% between LPSE-4 and LPSE-5. In LPSE-5 68% of paediatric LPS worked to a one-hour response time target to the ED. This is an increase from 42% (14/33) in LPSE-4.

**Conclusion:**

There are still far fewer paediatric than adult LPSs, but the provision of paediatric LPSs improved from 2015 to 2019, with more services, more staffing, and faster response times. Services need to continue to improve as few services match the adult core 24 criteria for an LPS.

